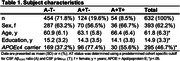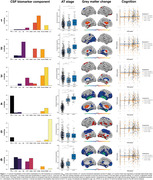# Neurobiological correlates of grey matter morphometry changes in preclinical Alzheimer’s disease

**DOI:** 10.1002/alz.091217

**Published:** 2025-01-09

**Authors:** Wiesje Pelkmans, Raffaele Cacciaglia, Gemma Salvadó, Karine Fauria, Carolina Minguillón, Gwendlyn Kollmorgen, Clara Quijano‐Rubio, Jose Luis Molinuevo, Oriol Grau‐Rivera, Rachael E Wilson, Erin M. Jonaitis, Rebecca E. Langhough, Kaj Blennow, Henrik Zetterberg, Sterling C. Johnson, Marc Suárez‐Calvet, Juan Domingo Gispert

**Affiliations:** ^1^ Barcelonaβeta Brain Research Center (BBRC), Pasqual Maragall Foundation, Barcelona Spain; ^2^ Hospital del Mar Medical Research Institute (IMIM), Barcelona Spain; ^3^ Centro de Investigación Biomédica en Red de Fragilidad y Envejecimiento Saludable (CIBERFES), Instituto de Salud Carlos III, Madrid Spain; ^4^ IMIM (Hospital del Mar Medical Research Institute), Barcelona Spain; ^5^ Clinical Memory Research Unit, Department of Clinical Sciences, Lund University, Lund Sweden; ^6^ Barcelonaβeta Brain Research Center (BBRC), Barcelona Spain; ^7^ Centro de Investigación Biomédica en Red de Fragilidad y Envejecimiento Saludable (CIBERFES), Madrid Spain; ^8^ Roche Diagnostics GmbH, Penzberg Germany; ^9^ Roche Diagnostics International Ltd., Rotkreuz Switzerland; ^10^ H. Lundbeck A/S, Copenhagen Denmark; ^11^ Hospital del Mar Research Institute (IMIM), Barcelona Spain; ^12^ Wisconsin Alzheimer's Disease Research Center, University of Wisconsin School of Medicine and Public Health, Madison, WI USA; ^13^ Department of Medicine, Division of Geriatrics and Gerontology, School of Medicine and Public Health, University of Wisconsin–Madison, Madison, WI USA; ^14^ Wisconsin Alzheimer’s Institute, University of Wisconsin School of Medicine and Public Health, Madison, WI USA; ^15^ Wisconsin Alzheimer’s Disease Research Center, University of Wisconsin School of Medicine and Public Health, Madison, WI USA; ^16^ Department of Psychiatry and Neurochemistry, Institute of Neuroscience and Physiology, The Sahlgrenska Academy, University of Gothenburg, Mölndal Sweden; ^17^ Clinical Neurochemistry Laboratory, Sahlgrenska University Hospital, Mölndal Sweden; ^18^ Hong Kong Center for Neurodegenerative Diseases, Hong Kong China; ^19^ Department of Neurodegenerative Disease, UCL Institute of Neurology, London United Kingdom; ^20^ Department of Psychiatry and Neurochemistry, Institute of Neuroscience and Physiology, The Sahlgrenska Academy, University of Gothenburg, Mölndal, Gothenburg Sweden; ^21^ Wisconsin Alzheimer’s Institute, University of Wisconsin‐Madison School of Medicine and Public Health, Madison, WI USA; ^22^ Wisconsin Alzheimer's Disease Research Center, Madison, WI USA; ^23^ Servei de Neurologia, Hospital del Mar, Barcelona Spain; ^24^ Centro de Investigación Biomédica en Red Bioingeniería, Biomateriales y Nanomedicina (CIBER‐BBN), Instituto de Salud Carlos III, Madrid Spain; ^25^ Hospital del Mar Research Institute, Barcelona, Barcelona Spain; ^26^ Universitat Pompeu Fabra, Barcelona Spain

## Abstract

**Background:**

The driving mechanisms of structural brain alterations in the earliest stages of Alzheimer's disease (AD) are not well understood. Previous heterogeneous findings in preclinical AD, including subtle atrophy and also increased grey matter (GM) volume, underscore the need for further exploration. This study uses an extensive fluid biomarkers panel to identify pathological drivers behind longitudinal GM changes in cognitively unimpaired (CU) adults.

**Method:**

We investigated 632 CU individuals (age 61.8±6.3, mean±SD) from the ALFA+, Wisconsin ADRC, and WRAP cohorts (Table 1), with available longitudinal MRI and cerebrospinal fluid biomarkers of Aβ_42/40_, p‐tau_181_, neurogranin, NfL, total‐tau, α‐synuclein, GFAP, YKL‐40, sTREM2, s100B, and IL‐6 measured using the NeuroToolKit panel of robust prototype assays (Roche Diagnostics International Ltd). We used non‐negative matrix factorization to decompose biomarkers into latent components. The per‐subject component weights were associated with voxel‐wise GM volume changes (years 3.5±0.9, mean±SD) and longitudinal performance on the preclinical Alzheimer's cognitive composite (PACC) using mixed model analysis.

**Result:**

Six biomarker components (C1‐C6) were identified (Figure 1). High expression of astrocyte biomarkers (C1) was related to GM volume increases in frontotemporal and subcortical regions, and faster cognitive decline in A+ individuals, while axonal damage with glial reactivity (C2) was associated with temporal atrophy. Microglial reactivity (C3) showed subtle associations with both increases and decreases in GM volume in different regions. A component primarily based on Aβ pathology (C4) was strongly associated with widespread GM volume loss and faster cognitive decline. IL‐6 (C5) reflected non‐AD related inflammation. Finally, the co‐expression of tau pathology, neuronal injury, and synaptic damage (C6) showed robust associations with frontotemporal atrophy and cognitive decline in A+ individuals.

**Conclusion:**

In a large longitudinal sample, we provide in vivo evidence that in the earliest stages of AD, Aβ pathology is also a strong contributor to future neuronal loss and cognitive decline besides neurofibrillary tangles and neuroaxonal damage. Astroglial reactivity (i.e. GFAP, s100B) in preclinical AD was associated with an increase in GM volume, which may suggest a neuroinflammatory response, and was associated with worse cognitive performance. These findings underscore the dynamic nature of structural brain changes within preclinical AD, shaped by the contributions from diverse pathophysiological processes.